# Automatic recognition of micronucleus by combining attention mechanism and AlexNet

**DOI:** 10.1186/s12911-022-01875-w

**Published:** 2022-05-18

**Authors:** Weiyi Wei, Hong Tao, Wenxia Chen, Xiaoqin Wu

**Affiliations:** 1 College of Computer Science and Engineering, Northwest Normal University, Lanzhou, China; 2 Radiology Department, Gansu Provincial Center For Disease Control And Prevention, Lanzhou, China

**Keywords:** Micronucleus, Computer-aided diagnosis, Convolutional neural networks, Visual attention, Data augmentation

## Abstract

**Background:**

Micronucleus (MN) is an abnormal fragment in a human cell caused by disorders in the mechanism regulating chromosome segregation. It can be used as a biomarker for genotoxicity, tumor risk, and tumor malignancy. The in vitro micronucleus assay is a commonly used method to detect micronucleus. However, it is time-consuming and the visual scoring can be inconsistent.

**Methods:**

To alleviate this issue, we proposed a computer-aided diagnosis method combining convolutional neural networks and visual attention for micronucleus recognition. The backbone of our model is AlexNet without any dense layers and it is pretrained on the ImageNet dataset. Two attention modules are applied to extract cell image features and generate attention maps highlighting the region of interest to improve the interpretability of the network. Given the problems in the data set, we leverage data augmentation and focal loss to alleviate the impact.

**Results:**

Experiments show that the proposed network yields better performance with fewer parameters. The AP value, F1 value and AUC value reach 0.932, 0.811 and 0.995, respectively.

**Conclusion:**

In conclusion, the proposed network can effectively recognize micronucleus, and it can play an auxiliary role in clinical diagnosis by doctors.

## Background

Micronucleus is a round-shaped fragment containing DNA, and it is separated from the nucleus during mitosis due to chromosome aberration caused by genotoxic and carcinogenetic agents [1, 2]. It has been shown that micronucleus may not only suggest the presence of problems, but also play a facilitating role in the process of genetic damage and tumour development. Therefore, computer-aided diagnostic systems for cell micronucleus are essential for detecting and treating tumours as well as DNA damages.

The in vitro micronucleus assay is at present used worldwide to detect whole chromosomes or chromosome fragments after nuclear division, allowing to identify the structural chromosome aberration [2, 3]. However, current clinical decision-making relies heavily on the expertise of physicians and researchers. Researchers have to use manual slide microscopy to enumerate micronuclei, which is tedious and error-prone. The excessive number of cases could stress physicians with the potential for misdiagnosis. Furthermore, staining the cells before diagnosis may contaminate the cells, making the visual scoring even more difficult.

In recent years, computer vision methods have succeeded in medical image analysis. It has advantages such as stability, standardization, long-term operation, and consistency [4]. Methods for diagnosis using computer vision are generally divided into traditional and deep learning techniques. Traditional methods design manual feature descriptors and feed the extracted features to the classifier for predictive results. For example, Mohammad et al. [5] firstly segmented the image and then performed micronucleus detection after processing the cell images using Nuc-Mask. However, the reliance of traditional methods on complex manual feature descriptor designs and image preprocessing limits the generality. Another kind of approach is based on convolutional neural networks (CNN). These methods implement training and testing end-to-end by feeding the original image into a deep learning network and outputting the prediction directly. Deep learning methods significantly improve classification accuracy and reduce the burden of designing manual feature descriptors [6]. Therefore, these methods are widely used and have succeeded in medical image classification tasks. For example, Alafif et al. [7] employed multiple transfer learning models for the classification of cell micronucleus images and then compared the results to obtain the optimal model. Chi et al. [8] proposed a CNN method combing deep and shallow features to detect thyroid nodule malignant risks in the ultrasound images. Work in [9] tried to use generative adversarial networks (GAN) to synthesize high-quality images of focal liver lesions from CT images, effectively alleviating the problem posed by the small dataset used for training. Many works focus on changing the structure of CNN itself. In [10], the author first proposed ELNet and dual-stream network (DSN) for segmentation and classification of esophageal lesion images. In [11], Gao et al. reported a dual-branch combinatorial network (DCN) for the joint segmentation and classification of covid-19 CT images. Wu et al. [12] proposed the covid-al framework, which can consider both data diversity and data uncertainty, improving the efficiency of active learning methods. In addition, Work in [4] fused neural networks and traditional methods, introducing the YOLO algorithm into cell micronucleus image detection, and achieved great performance.

**Fig. 1:**
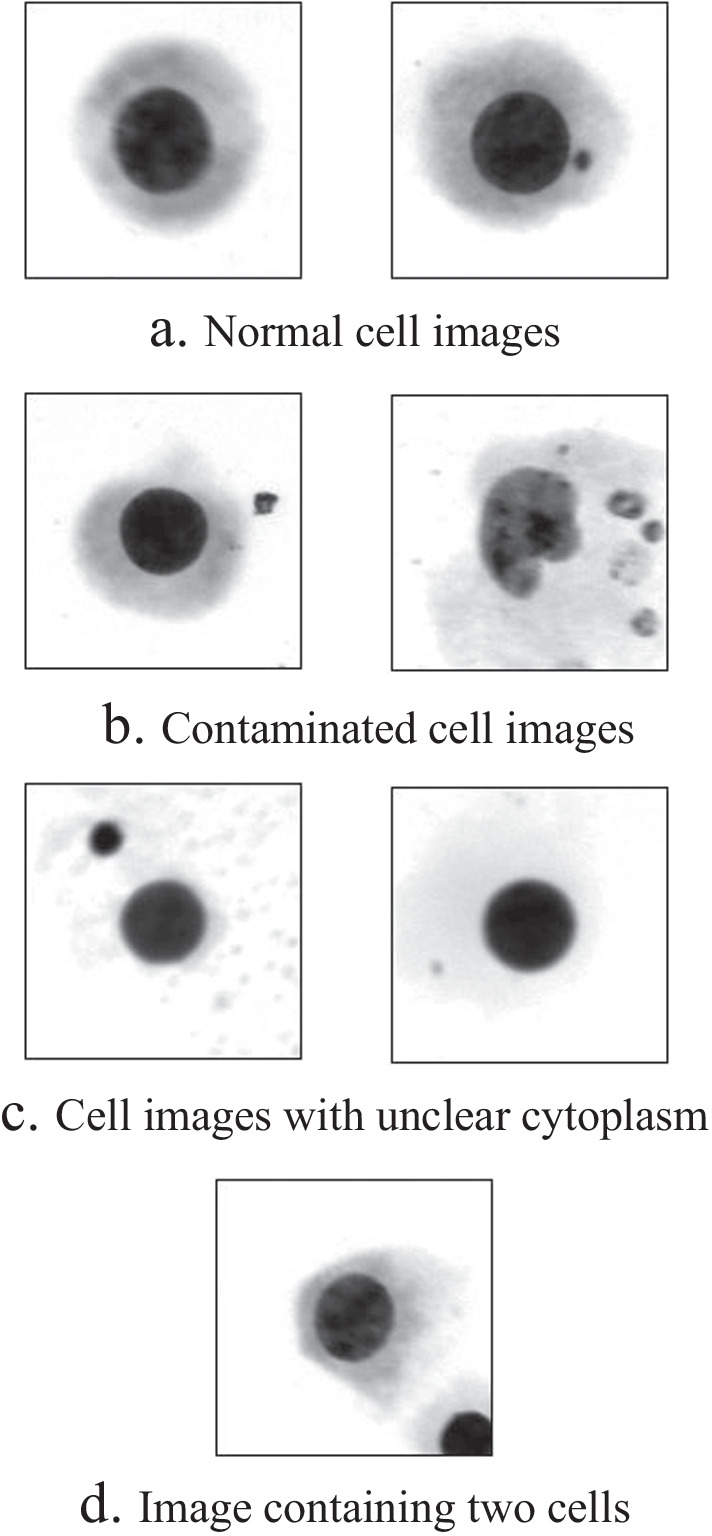
Examples of cell images

Despite the superior results of deep learning classification methods in medical diagnosis, the low-quality cell images in the dataset still make it a challenge to determine the presence of micronuclei using only cell images. Normal cell images (Fig. 1a) can help the model learn the boundaries of different classes of samples, but low-quality images may hinder the training process instead. Firstly, the staining process may contaminate the cells with black spots similar to micronuclei (Fig. 1b), causing the detection much harder. Secondly, the colour of the cytoplasm in the insufficiently stained cells was lighter (Fig. 1c), which also affected the judgment. Moreover, The presence of more than one cell in some images makes the tasks further difficult (Fig. 1d).

Due to the black-box nature of neural networks and the difficulty of producing outputs for specific pathological regions or lesion locations, there are significant interpretability problems, which makes CNNs detection results unconvincing. Since medical decision-making is related to physical health and even life safety, medical diagnostic applications not only require high performance but also require a strong judgment basis [13].

Based on the analysis above, to improve the accuracy and efficiency of cell micronucleus image detection and to mitigate the impact of the uninterpretability of neural networks and the complexity of the cell images themselves, we introduce an end-to-end convolutional neural network fusing AlexNet with the fully connected layer removed and visual attention. By using AlexNet with the fully-connected layer removed as the backbone network, the model parameters are significantly reduced, improving the efficiency of the network operation and reducing the possibility of overfitting. The attention maps generated by the attention module automatically highlight and display image regions relevant to the classification, thus developing interpretable information in addition to class labels. The network is implemented using the Pytorch framework and initialized using Alextnet pre-trained on ImageNet. Automatic detection is performed by fusing two attentional features and a depth feature to form a global feature.

## Methods

### Transfer learning

Transfer learning aims to improve the performance of a model in the current domain by transferring knowledge contained in a different but related field [14]. Deep transfer learning combines deep learning architecture and transfer learning. And the model is usually pre-trained on large-scale datasets such as ChEMBL and then fine-tuned on a specific dataset [15].

We use the AlexNet network as the backbone network and pretrain it for a good initialization. Transfer learning makes the network more lightweight and mitigates the possibility of overfitting. Because of the huge difference between ImageNet and our dataset, each layer of the network was retrained in the experiments.

### AlexNet

**Fig. 2:**
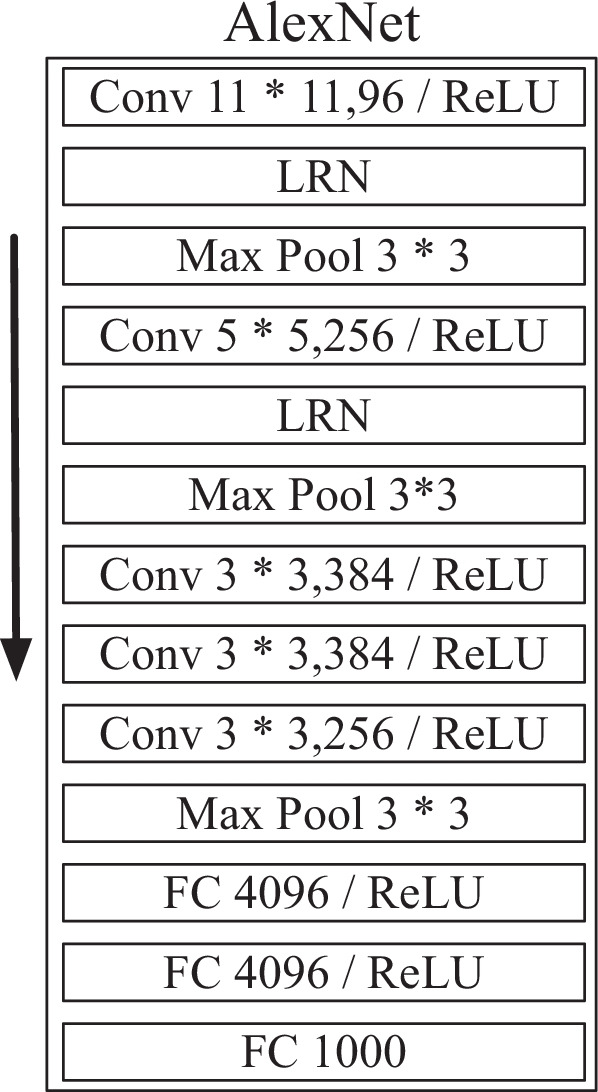
AlexNet network structure

AlexNet is one of the most famous convolutional neural network structures, which was proposed by Krizhevsky et al. [16]. AlexNet applies the ReLU activation function for solving the vanishing gradient problem, and the dropout technique is added to avoid overfitting by randomly deactivating some neurons. Although more advanced network models such as GoogLeNet [17] and ResNet [18] are available now, some researches show that for small multimodal medical image datasets, the classification results of GoogLeNet and AlexNet are very similar when rotation is used as the method of data augmentation. And for some categories, AlexNet even outperforms GoogLeNet. For newer networks such as VGG [19] and ResNet, the large number of parameters and complex structures mean a significant reduction in efficiency and a higher likelihood of overfitting. As the cell images used in this paper are not complex, consisting mainly of nuclei, micronuclei, cytoplasm, and a large amount of useless background information, Alextnet with fewer parameters is more efficient and sufficient to perform well on this classification task. Further, we remove the fully connected layer of AlextNet to improve our method’s efficiency. The structure of AlexNet is shown in Fig. 2.

### Attention mechanism

When looking at an object in the field of view, people focus on the parts of interest to them or more vital to problem solving. Specifically, when determining whether there are micronuclei in a cell image, we will focus on the micronuclei rather than other parts of the images. Attention mechanisms are proposed and embedded in convolutional neural networks to simulate this visual mechanism. Features of different image parts contribute differently to the overall classification task. The attention mechanism can automatically find and highlight the most informative parts of images to improve classification performance. Typically, this is achieved by generating an attention map of the original feature map.

Recently, attention mechanisms have been widely used in medical imaging diagnosis, and some new models have been proposed. Sun et al. [20] introduced a channel attention module for density classification in mammography. In [21], wang et al. trained a chest disease classification network incorporating a channel attention module, a scale attention module, and an element attention module. Work in [13] applied a diagnostic model for chest radiographs with global and local attention to improve the interpretability of convolutional neural network diagnosis.

Although many visual attention-based approaches have achieved promising results in the field of medical image analysis, the detection of micronuclei in cell images using features of a single layer in neural networks is still a challenge. Single-layer features contain limited information and only partially reflect the cell images. The attention module in our network produces more representative features by combining deep and shallow features. The fused features are used to generate a global feature fed to the classifier. This strategy alleviates the problem of single-layer features being too one-sided. Moreover, the attention module is independent and can be applied to different networks without modifying other parts of structures.

### Overall architecture

**Fig. 3:**
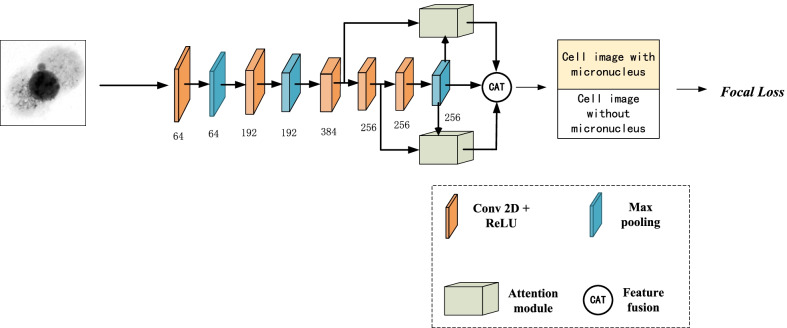
The overall network architecture

In this paper, AlexNet with fully connected layers removed is applied as the backbone network, and a spatial attention module is embedded in our model for interpretable information. Layer-5, layer-6 and the last layer (*L*) in Alexnet are used to compute the attention maps. Since the last layer is the deepest and most abstract layer in Alexnet and contains more semantic information, it serves as the lead feature when generating attention maps. Our model upsamples the feature *L* by means of bilinear interpolation and then feeds it to the attention block with the output of layer-5 and layer-6, respectively, to obtain the attention weight maps. Two attention maps are obtained by multiplying attention weight maps and the input feature. Finally, the global feature, formed by concatenating attention features and the input image, is fed into a softmax classification layer to obtain the classification result of cell images. The overall architecture of our network is illustrated in Fig. 3.

### Attention module

Let $M=\{ {M_1},{M_2} \cdots ,{M_C}\}$ and $L = \{ {L_1},{L_2}, \ldots ,{L_C}\}$ denote the output of the middle layer and last layers’ output, respectively. *C* is the number of channels, and $w \times w \times w$ is the size of the features. A 256-channel feature is obtained after bilinear interpolation and convolution, which is shown in Eq. (1).

1 $$\begin{aligned} {F_l} = bilinear({W_l} \otimes L) \end{aligned}$$

where ${W_l}$ is the weights of the convolution kernel corresponding to *L*, $\otimes$ is a convolutional operation, and $bilinear (\bullet )$ is the bilinear interpolation operation. The features of middle layers in the network are fed into a convolutional layer, yielding 256-channel outputs, which is shown in Eq. (2).

2 $$\begin{aligned} {F_m} = {W_m} \otimes M \end{aligned}$$

where ${W_m}$ is the weights of the convolution kernel corresponding to the middle layers of the network. ${F_l}$ and ${F_m}$ are fused to produce *F*, which is shown in Eq. (3).

3 $$\begin{aligned} F = W \otimes {\text {Re}} LU({F_l} + {F_m}) \end{aligned}$$

where ${\text {Re}} LU$ is the ReLU activation function, *W* is a convolution kernel that outputs a single channel. As in Eq. (4), The attention weight map is calculated by mapping the value of F to between 0 and 1 via the sigmoid activation function.

4 $$\begin{aligned} A = \sigma (F) \end{aligned}$$

where *A* and $\sigma ( \bullet )$ denote the attention weight map and the sigmoid activation function, respectively.

The attention feature map is obtained as follows:

5 $$\begin{aligned} \overline{{f_i}}= {a_i} \cdot f \end{aligned}$$

where $\overline{{f_i}}$ is the vector in attention feature map ${\overline{F}}$, $f_i$ and $a_i$ are the representations of the vector F and A, respectively. The features of layer-5 and layer-6 are fed into the attention module to compute the corresponding attention features. The global feature is obtained by concatenating two attention features and *L*, which is shown in Eq. (6).

6 $$\begin{aligned} {F_g} = cat(\overline{{F_5}} ,\overline{{F_6}} ,L) \end{aligned}$$

**Fig. 4:**
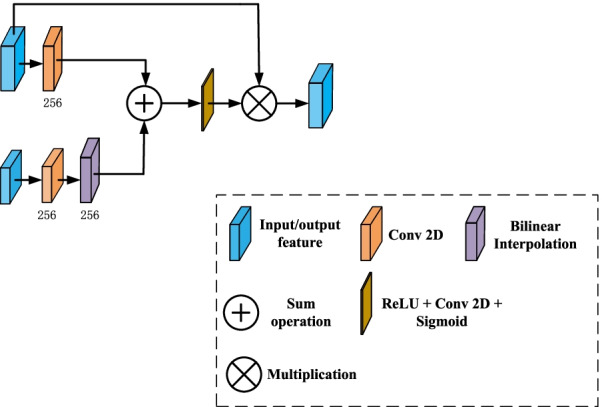
Attention module architecture

The architecture of the proposed attention module is presented in Fig. 4.

### Loss function

After generating the attention features in the middle layers, these features are concatenated with the output of the last layer in the network to obtain the global feature. Then, a classifier is trained based on this feature for final prediction. Because our dataset is small and extremely imbalanced, the network is trained using focal loss [22], an improved version of cross-entropy loss, to reduce the effect. Focal Loss achieves the purpose of focusing on hard-to-classify samples by reducing the weight of easy-to-classify samples. The cross-entropy is formulated as:

7 $$\begin{aligned} {L_{CE}}(p,y) = - [y\log (p) + (1 - y)\log (1 - p)] \end{aligned}$$

and formula for the cross focal loss is expressed as:

8 $$\begin{aligned} {L_{Focal}}(p,y) = - y{(1 - p)^\gamma }\log (p) - (1 - y){p^\gamma }\log (1 - p) \end{aligned}$$

where *y* and *p* represent the original label and predicted probability, respectively. The parameter $\gamma$ is used to reduce the loss of easily samples. When $\gamma$= 0, focal loss degenerates into the cross-entropy loss.We set $\gamma$ = 2 in experiements.

## Results

### Dataset and preprocessing

We use data from the Radiology Department of Gansu Provincial Center For Disease Control And Prevention in China to evaluate the performance of our model. All the images in our dataset, taken by the MetaSystems Metafer slide scanning platform, are individual lymphocytes (like Fig. 1 shows). Specialists have labelled them according to the presence of micronuclei. The dataset contains 726 cell images with micronuclei and 10419 cell images without micronuclei. This dataset is used to train a convolutional neural network for a cell images classification task. All images are resized to $224\times 224$ to fit the network.

### Evaluation metrics

We investigate the task of binary classification on cellular image data in this paper. We evaluate the classification performance of the network based on three metrics, including the area under the ROC curve (AUC), the average precision (AP), and the F1-score:

9 $$\begin{aligned} F1=\frac{2Precision*Recall}{Precision+Recall} \end{aligned}$$

where *Pecision* and *Recall* are defined as Eq. (10) and Eq. (11).

10 $$\begin{aligned} Precision&= \frac{TP}{TP+FP} \end{aligned}$$

11 $$\begin{aligned} Recall&= \frac{TP}{TP+FN} \end{aligned}$$

AUC values can be calculated using the true positive rate (TPR) and the false positive rate (FPR). It is an informative metric that can help avoid problems caused by imbalanced datasets. TPR and FPR are defined as follows:

12 $$\begin{aligned} TPR&= SEN \end{aligned}$$

13 $$\begin{aligned} FPR&= 1-SPE \end{aligned}$$

where

14 $$\begin{aligned} SEN&= \frac{TP}{TP+FN} \end{aligned}$$

15 $$\begin{aligned} SPE&= \frac{TN}{TN+FP} \end{aligned}$$

and *TP*, *TN*, *FP*, *FN* are the number of true positives, true negatives, false positives, and false negatives, respectively.

### Data augmentation

The dataset used in this paper is extremely imbalanced, which makes the network prefer the more frequent label. We perform the data augmentation strategy to address this issue, making our model more robust.

Network training aims to learn the boundary between images with and without micronuclei adequately, but this process can be affected by the imbalanced dataset. Therefore, the samples are first randomly divided into a training set, a validation set and a test set in a ratio of 6:2:2. Then, data augmentation is performed on the cell images with micronuclei, and we downsample the cell images without micronuclei to make the dataset tend to be balanced. The test set serves to evaluate the performance of the model using real samples, so no data augmentation is performed on it, and it is made to match the true sample distribution as closely as possible. The validation set also conforms to the true distribution. Experiments were conducted using the original validation set and the data-augmented validation set separately to assess the impact of data augmentation performed on the validation set when the images in the test set were all real data. The data set with only the training set augmented is denoted as TAD, and the data set with both the training and validation sets augmented is denoted as TVAD. The distribution of the number of images in the two data sets is shown in Tables 1 and 2.

In [23], the authors used random affine transformations (rotation, scaling, shearing and translation) as well as random elastic deformation for data augmentation. The images are first randomly rotated, then these rotated images are flipped horizontally, flipped vertically and scaled randomly. Finally, the amount of data reaches five times the original.

**Table 1:** Distribution of the dataset TAD

Dataset	Images without micronuclei	Images with micronuclei	Total
Training data	2191	2180	4371
Validation data	2084	145	2229
Test data	2083	145	2228

**Table 2:** Distribution of the dataset TVAD

Dataset	Images without micronuclei	Images with micronuclei	Total
Training data	2191	2180	4371
Validation data	10420	725	11145
Test data	2083	145	2228

### Experimental details

Our network initialized on ImageNet is implemented on Pytorch, a deep learning framework. We use the focal loss to alleviate the issue caused by imbalanced data. The network is trained for 60 epochs. During the training, the initial learning rate is 0.01 and decayed by 0.1 every 20 epochs. All the codes were run under Centos 7 with Intel(R) Xeon(R) Bronze 3106 CPU 1.70GHz, and RAM of 64GB.

We use the training subset to minimize the loss. During the training process, the checkpoint which maximizes the ROC value on the validation subset is saved, and we use it to evaluate the method performance on the test subset. This strategy is applied to all the compared models.

### Comparison of experiments on two datasets

The classification results and confusion matrices of the experiments on the two datasets are shown in Tables 3, 4,  5, respectively.

As shown in Table 3, our model achieves better performance on dataset TVAD (0.932, 0.811, 0.995 for AP, F1, and AUC, respectively). The confusion matrices show that there is more data clustered on the diagonal in Table 4. These results demonstrate that data augmentation on the validation set can improve classification performance. Therefore, the remaining comparison experiments are performed on the dataset TVAD.

**Table 3:** The experimental results of the proposed method on two data sets

Dataset	AP	F1	AUC
TAD	0.930	0.740	0.994
TVAD	**0.932**	**0.811**	**0.995**

The best results in this table are labeled in bold

**Table 4:** The confusion matrix of the proposed method on TVAD data set

Actual class	Predicted class	
	Image with micronuclei	Image without micronuclei
Image with micronuclei	137	8
Image without micronuclei	56	2027

**Table 5:** The confusion matrix of the proposed method on TAD data set

Actual class	Predicted class	
	Image with micronuclei	Image without micronuclei
Image with micronuclei	141	4
Image without micronuclei	95	1988

**Table 6:** Experimental results of different methods

Method	AP	F1	AUC
MobileNet [24]	0.589	0.504	0.931
VGG-16 [19]	0.868	0.803	0.989
VGG-Att	**0.948**	0.786	0.994
GoogLeNet [17]	0.871	0.780	0.988
GoogLeNet-Att	0.875	0.810	0.988
ResNet [18]	0.912	0.804	0.989
ResNet-Att	0.920	**0.877**	0.993
AlexNet [16]	0.824	0.749	0.984
Alex-light	0.875	0.800	0.990
MSA-Net [25]	0.919	0.808	0.989
Alex-CA	0.883	0.809	0.991
Our method-CE	0.818	0.548	0.985
Our method	0.932	0.811	**0.995**

The best results in this table are labeled in bold

### Comparison with classic networks

In this group of experiments, we compare our network with different classic models on the dataset TVAD (MobileNet, Vgg-16, GoogLenet, ResNet). The quantitive classification results are shown in Table 6. It can be observed that our method achieves higher performance than the baselines(0.932, 0.811, 0.995 for AP, F1, and AUC, respectively). Especially, the AP value of the proposed method is significantly better than other models. That is because 1. removing the dense layer of AlexNet makes our network more lightweight, which alleviates the overfitting issue. 2. attention module improves the classification performance.

### Comparison with other attention mechanisms

In this study [25], the author introduces the multi-scale attention network (MSA-Net) to enhance the discriminative power of the feature representation for DR classification. We replicate this network on our dataset to investigate the effectiveness of our method. Besides, we incorporate the channel attention from work [26] into Alex-light to design another variant (Alex-CA). It is equivalent to our network with the attention module replaced with a channel attention mechanism. The corresponding experimental results are presented in Table 6.

As shown in Table 6, our method outperform MSA-Net on micronucleus recognition task. The results show that scale attention does not perform well on our dataset, which may be since the scales of the parts in the cell pictures we used do not differ that much. Another observation is that although the channel attention mechanism improves the classification performance, our method obtains superior results with the same inputs.

### Ablation study

#### Effectiveness of the focal loss

To evaluate the effectiveness of the focal loss, we apply the cross-entropy loss version of our method, termed our method-CE, for a fair comparison. Referring to Table 6, replacing the focal loss with the cross-entropy loss makes the performance of our network get worse. This experiment indicates that even though the data augmentation has already been employed to make the training set almost ideally balanced, the focal loss could still be helpful for some latent reasons.

#### Comparison with the original AlexNet

Compared with the original AlexNet, the number of output nodes of our network is changed from 1000 to 2, we remove the fully connected layer, and an attention module is embedded. Referring to Table 6, our model still achieves better performance with fewer parameters than the original AlexNet.

#### Comparison with Alex-light

The reason why the proposed network model outperforms the original AlextNet may be that the original AlextNet contains too many parameters, leading to overfitting. We name the AlextNet network with the fully connected layers removed (it is equivalent to our network with the attention module removed) Alex-light and verify its performance on our dataset. According to Table 6, Alex-light slightly outperforms the original AlexNet, but the proposed network significantly outperforms the former two. This indicates that Alex-light mitigates the overfitting issue, but most of the superior performance of our network does not come from the elimination of the overfitting problem, but from the network structure itself.

#### Comparison with VGG-Att, GoogLeNet-Att and ResNet-Att

**Fig. 5:**
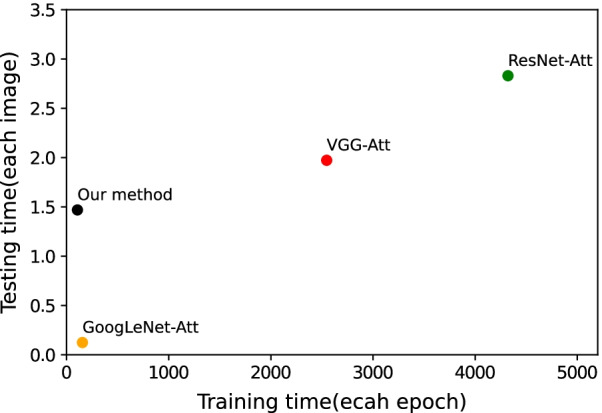
Training and testing time that is in second

The experimental results (Table 6) show that VGG-16, GoogLeNet, and ResNet outperform AlexNet on our dataset. For a fair comparison, we apply the modifications made for AlexNet to these three more advanced architectures and evaluate the impact. We truncate the dense layers of VGG-16 (GoogLeNet and ResNet use global average pooling to process the final convolutional feature map instead of the dual-stacked fully-connected layers for fewer parameters) and incorporate the attention mechanism into these three networks. The new architectures are termed VGG-Att, GoogLeNet-Att, and ResNet-att, respectively. The experimental results are added to Table 6. We can observe that the modifications make the networks achieve better performance. The AP value of VGG-att and the F1 score of ResNet-Att are higher than those of our method. However, more advanced architectures such as VGG and Resnet always come with more parameters and complex strategies that make the training and classification extremely time-consuming. It could be a problem because one single cell image contains such limited information that we need to examine vast amounts of them to provide a solid basis for clinical diagnosis. The training and testing time of these models on our dataset are depicted in Fig. 5. As shown in Fig. 5, ResNet-Att and VGG16-Att are significantly less efficient than GoogLeNet-Att and our method, consistent with the previous description. These results demonstrate that our network strikes a good balance between efficiency and classification performance, which validates that lightweight networks are sufficient to perform well on our dataset.

### Visualization of attention features

**Fig. 6:**
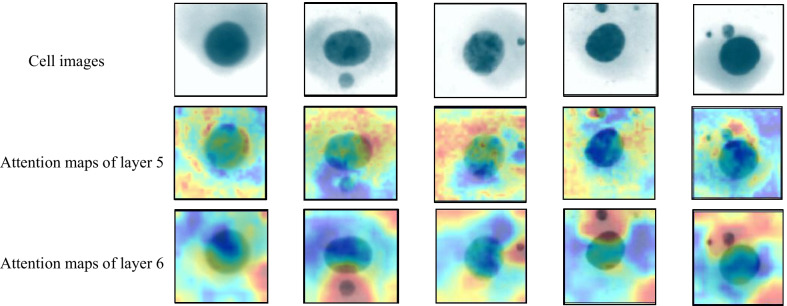
Visualization of attention maps

To verify whether the superior performance implies better visual interpretability, we upsample and visualize the attention feature maps of middle layers in the network. As shown in Fig. 6, the feature maps highlight regions highly relevant to the diagnosis.

It can be observed from Fig. 6 that the attention maps of the deep layer(layer 6)accurately highlight the micronucleus, but the shallow attention maps(layer 5)do not seem to learn any useful information. This may be because deep layers in convolutional neural networks usually focus on more abstract information than shallow layers, ignoring parts not relevant to the detection task.

## Discussion and conclusion

In this paper, we propose an attention-based network with an explanation, which is one of the only few attempts using an interpretable model to detect micronucleus in cell images. We remove the dense layer of our network and pretrain it on ImageNet, which makes the network run more efficiently. Moreover, data augmentation is applied to mitigate the over-fitting risk.

The experiments are first conducted on two datasets to demonstrate the effectiveness of data augmentation on the validation set. We compare our model with several classic networks, and the evaluation shows that our model achieves better performance.

There are two directions for further works. The first is improving the network structure to increase the interpretability of the diagnostic method. The second is extending our approach to multiclassification problems to detect the number of cell micronuclei accurately.

## Data Availability

The datasets generated and/or analysed during the current study are not publicly available due to the regulations, but are available from the corresponding author on reasonable request.

## Abbreviations

MN: Micronucleus
CNN: Convolutional neural networks
ROI: Region of interest
AP: Average precision
AUC: Area under curve
GAN: Generative adversarial networks
DSN: Dual-stream network
DCN: Dual-branch combinatorial network
TPR: True positive rate
FPR: False positive rate
